# A commentary on ‘Whether surgical procedure can improve the prognosis of endometrial cancer arising in adenomyosis (EC-AIA)? A systematic review and meta-analysis’

**DOI:** 10.1097/JS9.0000000000001435

**Published:** 2024-04-09

**Authors:** Liming Zhou, Mai Li, Jie Li

**Affiliations:** Reproductive Medicine Center, Women and Children’s Hospital of Ningbo University, Zhejiang, People’s Republic of China

Endometrial cancer (EC) is the sixth most detected cancer and the 14th most prevalent cause of cancer death in women globally, affecting 2.8% of women at some point in their lifetime^1^. Patients typically present with uterine-confined pathology and have high survival rates. The key predictors of cancer outcome are histological character, tumor grade and size, age, degree of myometrial invasion, lymph node involvement, and disease stage.

One of the most prevalent pathological signs in hysterectomy tissues is known as adenomyosis, which spreads from the endometrium into the uterus, the myometrium, and the endometrial glands. With a varying prevalence of 12–66%^2^, it is one of the most common ancillary histopathological results of EC, especially of the endometrioid histotype. Numerous studies have investigated whether adenomyosis is present in EC patients. Those who investigated the importance of adenomyosis in endometrial adenocarcinoma suggested that it had negative impacts on EC. However, in other studies, adenomyosis with EC has been linked to early-stage malignancy and extended surveillance. Furthermore, subsequent investigations have demonstrated that adenomyosis does not negatively affect the prognosis and questionnaires of EC patients. It is therefore still unclear whether EC and adenomyosis are related.

Sun *et al*.^3^ performed a systematic review and meta-analysis to explore the impact of lymphadenectomy on the prognosis of EC arising in adenomyosis (EC-AIA). The results of the study indicated that for patients who are unexpectedly diagnosed with EC-AIA following hysterectomy for benign tumors, a second surgery of supplementary lymphadenectomy may be a better option.

Koshiyama *et al*.^4^ reported only four cases in 564 patients (0.74%) operated between 1981 and 2001. Although the exact cause of malignant transformation in adenomyosis is still unknown, several writers have suggested that genetic and epigenetic factors may be involved. Due to the absence of an anatomic set in the basal part of endometrial tissue, cancer first develops within the myometrial part and smoothly spreads to the myometrial stromal layer. Cancer that has directly invaded the myometrial stromal tissue extends rapidly to the lymphatic and circulatory systems. However, many molecular elements of the malignant development of adenomyosis remain unknown. The relationship between adenomyosis and the disruption of heterozygosity in the DNA mismatch repair gene has only been briefly described in research^5^.

The present research has some drawbacks. First, there is a deficiency of pathological evaluation to differentiate between ECs with adenomyosis and ECs developing from adenomyosis foci. These two disorders are histopathologically and clinically diverse, with different diagnostic criteria and biological characteristics. Second, this study did not allow us to assess polymerase epsilon (POLE) mutations. We think that one of the crucial conditions for upcoming research on EC is the examination of the POLE mutation. Finally, this article was a retrospective study, as further prospective studies are still needed for validation.

## Ethical approval

Not applicable.

## Consent

Not applicable.

## Sources of funding

1.Ningbo Science and Technology Project (2023Z178).

2.Ningbo Clinical Medical Research Center (2024L002).

3. Ningbo Key Laboratory for the Prevention and Treatment of Embryo Original Diseases.

## Author contribution

L.Z.: wrote the paper; M.L.: data analysis; J.L.: study design.

## Conflicts of interest disclosure

There are no conflicts of interest.

## Research registration unique identifying number (UIN)

Not applicable.

## Guarantor

Liming Zhou.

## Data availability statement

Not applicable.

## Provenance and peer review

Not applicable.
